# Research progress on the role of exercise-regulated reactive astrocytes in the prevention and treatment of Parkinson’s disease

**DOI:** 10.3389/fnagi.2025.1561006

**Published:** 2025-06-05

**Authors:** Quan Yang, Chunyu Zhuang

**Affiliations:** ^1^School of Sports Science, Jishou University, Jishou, China; ^2^Haikou Maternal and Child Health Hospital, Haikou, China

**Keywords:** exercise, Parkinson’s disease, reactive astrocytes, neuroinflammation, physical activity

## Abstract

Astrocytes generally perform protective roles, such as the release of neurotrophic factors, glutamate metabolism, transfer of healthy mitochondria to neurons, and maintenance of the blood-brain barrier. Nonetheless, in the context of Parkinson’s disease (PD), astrocytes may become dysfunctional, contributing to neurotoxicity, which is intricately linked to the etiological factors of PD. Intervening to prevent the conversion of astrocytes into neurotoxic phenotypes has demonstrated neuroprotective effects, potentially averting the degeneration of dopaminergic neurons and mitigating behavioral deficits in PD model mice. Research has shown that exercise decreases the prevalence of central pro-inflammatory and neurotoxic reactive astrocytes while increasing the presence of anti-inflammatory and neuroprotective reactive astrocytes. Various forms of exercise therapy are extensively employed as adjunctive treatments alongside pharmacotherapy in PD patients, and have been empirically validated to directly enhance motor function, functional flexibility, gait, balance, fine motor skills, and overall quality of life in individuals with PD. The potential mechanism of various types of exercise therapy in improving PD-related behavioral dysfunction is closely related to the regulation of the conversion of pro-inflammatory and neurotoxic reactive astrocytes to anti-inflammatory and neuroprotective astrocytes by exercise. This paper discusses the regulatory role of reactive astrocytes in neuroinflammation and PD neurodegeneration, as well as the reduction of neuroinflammation and the progression of PD through exercise regulation of reactive astrocytes, so as to provide a theoretical basis for further exploring the pathogenesis of PD and further developing therapeutic interventions for neurodegenerative diseases.

## Introduction

Astrocytes are the most abundant cells in the central nervous system (CNS) and are involved in maintaining its physiological functions. Under physiological conditions, astrocytes have various important functions such as pH, ion and redox buffering, as well as blood flow, neurotransmitter circulation, and energy homeostasis regulation (Linnerbauer et al., 2020). When the body is subjected to pathological stimuli such as inflammation, trauma, cerebral ischemia, and neurodegeneration, astrocytes will undergo morphological and molecular changes, forming reactive astrocytes (Ben Haim et al., 2015). It not only exacerbates the amplification of inflammatory signals, but also generates glutamate and oxidative stress products, mediating the death of dopamine (DA) neurons (Morales et al., 2016; Zhu et al., 2020). Furthermore, as an important component of the blood-brain barrier (BBB), astrocytes activate matrix metalloproteinases (MMPs), disrupt the basement membrane, increase BBB permeability, and cause peripheral immune cells to infiltrate the brain matrix, further exacerbating the inflammatory response and death of dopaminergic neurons during the development of Parkinson’s disease (PD) (Liu et al., 2022). Currently, the pathogenic mechanisms of PD are not fully understood. Many hypotheses suggest that several factors are related to PD, including the α-synuclein aggregation, dopaminergic neuron loss, autophagy lysosomal system dysfunction, mitochondrial damage, vesicle transport defects, dysbiosis of the gut microbiota, oxidative stress, and glutamate excitotoxicity (He et al., 2020). However, numerous pieces of evidence indicate that activated reactive astrocytes and reactive astrocyte-mediated neuroinflammation play an important role in the pathological process of PD, participate in its initiation and progression (Lee et al., 2022). Research has shown that misfolded α-synuclein is released from the axon terminals of neurons and accumulates in the cytoplasm of astrocytes, causing them to produce pro-inflammatory factors such as INF-γ and TNF-α, leading to astrocyte activation and dysfunction. Activated reactive astrocytes can cause the loss of dopaminergic neurons by disrupting the glutamate cycle, potassium buffering, and calcium signaling. In addition, reactive astrocytes have been show to interact with microglia to jointly promote the inflammatory cascade in PD (Takahashi and Mashima, 2022). Research has confirmed that directly preventing the transformation of astrocytes into a pro-inflammatory neurotoxic phenotype mediated by microglia has neuroprotective effects, which can prevent the loss of dopaminergic neurons and behavioral defects in the midbrain of sporadic Parkinson’s disease α-synuclein prefabricated fiber (α-syn PFF) mouse models (Zhang et al., 2025). Specifically, the number of TH immunopositive cells in the substantia nigra significantly increased, the concentration of DA in the striatum significantly increased, and the number of rotations induced by apomorphine APO and the time required for pole climbing significantly decreased (Yun et al., 2018). Studies have shown that exercise can significantly downregulate the expression levels of central glial fibrillary acidic protein and complement component 3 (pro-inflammatory astrocyte biomarker) protein, as well as interleukin-1β (IL-1β) and tumor necrosis factor-α (TNF-α) mRNA (Wang et al., 2024; Kelty et al., 2022). The study also confirmed that regardless of the intensity and mode of exercise, it can increase the number and activity of anti-inflammatory glial cells in the central nervous system of exercised mice (Eo and Leem, 2023). Various types of exercise therapy are widely used as adjunctive therapy for drug therapy in PD patients, and have been proven to have direct beneficial effects in improving PD-related behavioral impairments such as motor function, functional flexibility, gait, balance, fine motor function, and quality of life (Langeskov-Christensen et al., 2024). The potential mechanisms of various types of exercise therapy for improving PD-related behavioral dysfunction are closely related to neuroinflammation mediated by exercise-regulated reactive astrocytes. This article discusses the regulatory role of reactive astrocytes in neuroinflammation and PD neurodegenerative diseases, as well as the use of exercise to reduce neuroinflammation and alleviate PD progression by regulating reactive astrocytes. This provides a theoretical basis for further exploration of the pathogenesis of PD and development of therapeutic interventions for neurodegenerative diseases.

## Reactive astrocytes

Astrocytes are essential for maintaining homeostasis of the Central Nervous System (CNS) and dynamically respond to injury or disease. In terms of development, astrocytes originate from pressure-controlled synchronized intermittent mandatory ventilation plus pressure support ventilation (NPCs) in the subventricular zone (SVZ), and participate in maintaining the physiological functions of the CNS by migrating along radial glial processes to fill the entire brain (Ge et al., 2012; Moyon et al., 2015). During neuroinflammation, astrocytes undergo reactive reactions, that are characterized by morphological changes and upregulation of glial fibrillary acidic protein (GFAP). In their reactive state, pro-inflammatory astrocytes secrete a variety of pro-inflammatory cytokines, such as IL-1β, TNF-α, and interferon-γ (IFN-γ). These cytokines can activate microglia and other immune cells, thereby exacerbating neuroinflammation. In addition, the matrix metalloproteinases [such as Matrix metalloproteinase (MMP-2) and (MMP-9)] secreted by pro-inflammatory astrocytes can degrade components of the extracellular matrix (ECM) and basement membrane, such as collagen, laminin, and fibronectin, compromising the integrity of the blood-brain barrier (BBB). This, in turn, facilitates the invasion of tumor cells and infiltration of inflammatory cells. This process can further lead to pro-inflammatory astrocytes that release harmful toxic substances posing a threat to oligodendrocytes and neurons, thereby affecting the behavior of the surrounding neurons and immune cells (Ding et al., 2021). Moreover, neuroinflammation triggered by the overactivation of astrocytes appears to converge on certain common downstream transcriptional regulatory factors (Vitorino et al., 2022). The activation of nuclear factor kappa-B (NF-κB) and its translocation to the nucleus are key links in the activation of astrocytes, and the nuclear translocation of NF-κB is finely regulated by multiple complex mechanisms. The nuclear migration mechanism of NF-κB in astrocytes is triggered by a series of pro-inflammatory stimuli, such as interleukin-1β (IL-1β), tumor necrosis factor-α (TNF-α), and IL-17 release, as well as other factors related to CNS inflammation (Linnerbauer et al., 2020). After IL-17 is released and binds to its receptor, it activates NF-κB through adaptor proteins such as actin-1 (ACT1) and TNF receptor-associated factor 6 (TRAF6), which in turn promotes the expression of various pro-inflammatory cytokines and chemokines, thereby playing an important role in inflammatory responses(Kwon and Koh, 2020). In addition, sphingosine-1-phosphate (S1P) derived from ceramides is involved in driving the activation of NF-κB and plays a key role in the pathological regulation of PD and Alzheimer’s disease (AD) (Czubowicz et al., 2019). Research has shown that astrocyte activation can be inhibited by suppressing the increase in nitric oxide induced by S1P and inhibiting the activation of the NF-κB signaling pathway (Lee et al., 2020). Lactosylceramide (LacCer) is another sphingolipid derived from ceramides, that also participates in the regulation of astrocyte responses during CNS inflammation. LacCer synthesis is catalyzed by beta-1,4-galactosyltransferase 6 (B4GALT6), whose expression is controlled by NF-κB and upregulated in astrocytes in experimental autoimmune encephalomyelitis (EAE) in non-obese diabetic mice (Mayo et al., 2014). Based on the potential harm caused by abnormal activation of NF-κB, there are multiple mechanisms that limit the activation of NF-κB in astrocytes. One mechanism involves ligand-activated transcription factor aryl hydrocarbon receptors (AHRs), the activity of which is regulated by small molecules provided by the cell and symbiotic microbiota metabolism (Lee et al., 2022). After activation by its agonists, AHR can restrict NF-κB signaling through various mechanisms, including the mechanism mediated by suppressor of cytokine signaling 2 (SOCS2) and the direct dimerization of AHR with NF-κB subunits RelA and RelB (Linnerbauer et al., 2020). Tryptophan is an essential amino acid. Its metabolites, such as indole-3-acetic acid (IAA) and indole-3-aldehyde (IAM), can activate the aryl hydrocarbon receptor (AhR), thereby inhibiting pro-inflammatory astrocyte signaling pathways, such as the NF-κB signaling pathway, and reducing the production of inflammatory cytokines, such as IL-1β, thus exerting anti-inflammatory effects. Therefore, dietary metabolites derived from tryptophan inhibit NF-κB signaling in astrocytes and limit CNS inflammation in an AHR-dependent manner (Rothhammer et al., 2016). Specific inactivation of AHR in astrocytes exacerbates EAE and increases the expression of pro-inflammatory cytokines (IL-6, IL-12, IL-23, GM-CSF, and NO), chemokines (CCL2, CCL20, CXCL10), and other molecules GFAP associated with astrocyte reactivity.

## Reactive astrocytes and the occurrence and development of PD

Inflammatory cytokines (such as TNF-α, IL-1β, and IL-6) are closely related to the occurrence and development of PD (Wang T. et al., 2023). DAergic neurons appear to be particularly sensitive to inflammatory cytokines. Studies have shown that TNF-α expression in the mouse brain limits the number of TH-immunoreactive cells in the caudate-putamen and affects grooming behavior in mice (Aloe and Fiore, 1997). Alterations in grooming behavior suggest that TNF-α affects neurons, and the reduction in TH-immunoreactive cells can be interpreted as a consequence of TNF-α-induced DAergic neuron loss. This reduction in TH corresponds to a decrease in DA levels, which underlies many of the symptoms observed in PD.

Astrocytes have been implicated in the production and release of inflammatory cytokines, which can exacerbate neuroinflammation and neurodegeneration in PD. The first pathway involves reactive microglia, which are present in significant numbers in patients with PD. Reactive microglia have been shown to release various inflammatory cytokines, such as IL-1α, leading to the transformation of astrocytes into a pro-inflammatory phenotype. Reactive neurotoxic astrocytes increase the expression of pro-inflammatory cytokines, thereby amplifying neurotoxicity. Compounding this issue is the fact that astrocytes do not release neurotrophic factors or antioxidants. Postmortem brain examinations of patients with PD have confirmed that up to 40% of astrocytes in the striatum can be pro-inflammatory, and α-synuclein (α-Syn) can directly or indirectly activate pro-inflammatory microglia, which in turn prompts astrocytes to shift to a pro-inflammatory state. The substantial release of pro-inflammatory factors such as TNF-α, complement 3 (C3), and IL-1β by these astrocytes exacerbates neuroinflammatory and immune cascade reactions (Wang J. et al., 2023). Studies by Miyazaki and Asanuma (2020) have shown that the upregulation of pro-inflammatory astrocytes in the brains of PD patients is closely related to the degeneration and loss of DAergic neurons in the substantia nigra pars compacta of the midbrain, the depletion of extracellular DA in the striatum, and the abnormal accumulation of α-Syn in the cytoplasm of the remaining DAergic neurons, forming large amounts of eosinophilic inclusions known as Lewy bodies. Liddelow et al. (2017) demonstrated that activated microglia prompt pro-inflammatory astrocytes to enhance the expression of pro-inflammatory factors (IL-1α, IL-1β, and TNF-α), thereby causing astrocytes to lose their original ability to promote neuronal survival, growth, synapse formation, and phagocytic functions. In recent years, Joshi et al. (2019) also confirmed that mitochondrial fragments released by microglia can trigger the response of pro-inflammatory astrocytes, thereby initiating inflammatory neurodegenerative processes. These observations indicate that microglia play a key role in stimulating astrocytes to produce inflammatory responses. However, there are reports that astrocytes can regulate the activation state of microglia and affect the neuroinflammatory process mediated by microglia (Jo et al., 2017). This finding highlights the central role of the complex interplay between astrocytes and microglia in the development of neuroinflammation.

The nucleotide-binding oligomerization domain-like receptor protein 3 (NLRP3) inflammasome is a core regulatory factor in neuroinflammation (Guan and Han, 2020). Activation of the NLRP3 inflammasome in microglia and astrocytes, induces the release of pro-inflammatory cytokines IL-1β and IL-18 (Guan and Han, 2020). Studies have found that the concentrations of IL-1β and IL-18 in the cerebrospinal fluid of patients with PD are significantly higher than those in healthy individuals, further confirming their role in the pathological process of PD (Freeman et al., 2017). In addition, inhibiting the activation of the NLRP3 inflammasome in astrocytes and the subsequent production of IL-1β has been found to have neuroprotective effects, confirming the central role of these cytokines in the pathogenesis of PD (Pike et al., 2022). In addition to the NLRP3 inflammasome, the NF-κB signaling pathway in astrocytes can also induce neuroinflammation. Studies have shown that in a PD animal model induced by the neurotoxin 1-methyl-4-phenyl-1,2,3,6-tetrahydropyridine (MPTP), astrocytes mediate inflammatory responses through the NF-κB signaling pathway, which is one of the key factors leading to DAergic neuron damage (Kirkley et al., 2019; Hammond et al., 2020). This confirmed the multiple roles of astrocytes in neuroinflammation and neuronal damage in PD (Table 1). In addition, researchers have observed blood–brain barrier (BBB) leakage in brain regions associated with PD (such as the basal ganglia) in PD patients (Lau et al., 2024). This suggests that BBB disruption may be involved in PD development. As astrocytes are crucial for the BBB, astrocyte dysfunction may be a major cause of BBB disruption. Studies have shown that astrocytes promote the secretion of tight junction proteins through the production of growth factors.

**TABLE 1:** Expression levels of inflammatory cytokines mediated by astrocytes in PD state.

Authors	PD patients or PD animal models	The expression level of inflammatory factors mediated by reactive astrocytes
Wang L. et al. (2023)	PD patients	striatumα-Syn↑, TNF-α↑, C3↑ and IL-1β↑
Miyazaki and Asanuma (2020)	6-OHDA induced PD model mice	Pro-inflammatory astrocytes in the striatum↑ , α-Syn↑ , DA↓
Liddelow et al. (2017)	LPS induced PD model	Dorsal lateral geniculate nucleus IL-1α↑ , TNFα↑ , C1q↑ ; pro-inflammatory astrocytes↑
Joshi et al. (2019)	MPTP induced PD animal model	Hippocampus and striatum GFAP↑ , Iba-1↑ ,TNFα↑, IL-6↑ ,IL-1α↑ ,IL-1β↑
Pike et al. (2022)	6-OHDA induced PD model mice	NLRP3↑ , IL-1β↑ , DA↓
Hammond et al. (2020)	MPTP induced PD animal model	NF-κB↑ , DA↓, GFAP^+^↑ of SN

However, in PD, reactive astrocytes have been shown to significantly reduce the expression of growth factors, which in turn decreases the production of tight junction proteins and leads to loss of BBB integrity. Compounding this issue is the fact that reactive astrocytes express VEGF-A, which downregulates the tight junction proteins claudin-5 and occludin (Khor et al., 2024). Loss of BBB integrity may lead to neuronal death by making the brain more susceptible to environmental toxins or other insults.

## Prevention of astrocyte conversion into a pro-inflammatory phenotype and inflammatory response in the prevention and treatment of Parkinson’s disease

Pro-inflammatory astrocytes secrete many inflammatory substances that can damage neurons. In PD, the conversion of astrocytes to a pro-inflammatory phenotype can be attributed to activated microglia. Therefore, inhibiting the conversion of astrocytes into a pro-inflammatory phenotype seems to be a promising strategy for the prevention and treatment of PD, although further research is needed to fully understand the underlying mechanisms and develop effective therapeutic strategies. Chung et al. (2017) explored the effects of capsaicin (CAP), a transient receptor potential vanilloid subtype 1 (TRPV1) agonist, on nigrostriatal dopaminergic neurons in MPTP-induced PD model mice. The results indicated that CAP, by activating TRPV1, inhibited the activation of microglia and the conversion of astrocytes into a pro-inflammatory phenotype, thereby reducing the expression levels of pro-inflammatory cytokines (tumor necrosis factor-α and interleukin-1β) and the production of reactive oxygen/nitrogen species derived from activated microglia (e.g., NADPH oxidase, inducible nitric oxide synthase) or pro-inflammatory astrocytes (e.g., myeloperoxidase). This led to neuroprotective effects, with significant reductions in nigrostriatal dopaminergic neuronal loss in MPTP-treated mice, significant increases in extracellular dopamine levels in the striatum, and marked improvements in behavioral dysfunction, as evidenced by a significant increase in the time the mice could maintain balance on a rotating rod. The anti-cholesterol drug simvastatin has been found to have neuroprotective effects in neurotoxin models both *in vivo* and *in vitro*, partly because it can prevent astrocytes from converting into a neurotoxic phenotype (Du and Bu, 2021). Tong et al. (2018) demonstrated that the cell viability of SH-SY5Y cultures treated with simvastatin and 6-OHDA was 59.58 ± 5.80% at 24 h, whereas that of SH-SY5Y cultures treated solely with 6-OHDA was only 47.34 ± 7.40%. Activation of the nicotinamide adenine dinucleotide phosphate (NADPH) oxidase/p38 mitogen-activated protein kinase (MAPK) pathway was inhibited, and nuclear transcription of nuclear factor-κB (NF-κB) was reduced in SH-SY5Y cells following simvastatin treatment. *In vivo* studies revealed that the administration of simvastatin by gavage decreased limb-use asymmetry and apomorphine-induced rotations in 6-OHDA-lesioned mice. Simvastatin increased the survival of dopaminergic neurons in the midbrain of PD mice and reduced tyrosine nitration and gliosis, with an inhibitory effect on the activation of NADPH oxidase/p38 MAPK and increased expression of antioxidant proteins in the midbrain. Bear bile powder (BBP) is a traditional Chinese medicine with anti-inflammatory and other properties. Wang T. et al. (2023) explored the role and mechanism of BBP in suppressing astrocyte-mediated neuroinflammation in a PD mouse model. The results showed that BBP treatment significantly improved motor dysfunction in MPTP-induced PD model mice, upregulated the expression of tyrosine hydroxylase (TH), and inhibited the overactivation of astrocytes in the substantia nigra (SN) of PD mice. In addition, BBP reduced the protein levels of glial fibrillary acidic protein (GFAP), cyclooxygenase 2 (COX2), and inducible nitric oxide synthase (iNOS), and upregulated the levels of TGR5 protein in the SN. Moreover, BBP significantly activated Takeda G-protein receptor 5 (TGR5) in a dose-dependent manner, both *in vivo* and *in vitro*, and reduced the protein levels of GFAP, iNOS, and COX2, as well as the mRNA levels of GFAP, iNOS, COX2, interleukin (IL)-1β, IL-6, and tumor necrosis factor-α (TNF-α) in LPS-stimulated C6 cells. Notably, BBP inhibited the phosphorylation of protein kinase B (AKT), NF-κB inhibitor (IκBα), and nuclear factor-κB (NF-κB). Morin is a flavonol with antioxidant and anti-inflammatory properties that is found in wine, herbs, and fruits. Hong et al. (2022) found that dietary morin significantly improved motor dysfunction in MPTP-induced PD model mice and significantly reduced dopaminergic neuronal damage in the striatum (STR) and substantia nigra (SN) of PD model mice. In addition, morin significantly reduced the expression levels of TNF-α, IL-6, and IL-1β in the striatum of MPTP-induced PD model mice and effectively inhibited the activation of microglia and astrocytes. Studies have shown that the Atp13a2 (Park9) gene encodes a transmembrane lysosomal P5-type ATPase (ATP13A2), and its missense or truncation mutations lead to lysosomal dysfunction, which in turn causes neuronal death during the pathogenesis of Parkinson’s disease (PD) (Croucher and Fleming, 2023). Qiao et al. (2016) confirmed that the loss of ATP13A2 in astrocytes triggers a robust inflammatory response, exacerbating dopaminergic neuronal damage following MPP + exposure. Notably, the absence of ATP13A2 increases lysosomal membrane permeability and the release of cathepsin B, thereby enhancing activation of the NLRP3 inflammasome and leading to excessive IL-1β production by astrocytes. Moreover, overexpression of ATP13A2 reversed the release of cathepsin B and activation of the NLRP3 inflammasome induced by MPP + in astrocytes.

In summary, strategies to prevent astrocytes from converting into a pro-inflammatory phenotype and mitigating inflammatory responses may serve as effective means for PD treatment. However, the aforementioned studies have focused on inhibiting astrocytes via drugs and other neuroprotective agents; yet, precise activation and targeted drug delivery remain challenging, thus limiting clinical interventions. Future research should focus on manipulating astrocytes through various approaches such as optogenetics and chemogenetics, which would allow for more refined interventions in mechanisms related to neuroinflammation and facilitate targeted intervention of the specific mechanisms of neuroinflammation mediated by astrocytes in PD.

## The effect of exercise on reactive astrocytes

Studies have shown that exercise has a regulatory effect on reactive astrocyte-mediated neuropathy. Three months of aerobic exercise training enhanced the expression of antioxidant factors in animal brains. However, prolonged exercise until exhaustion can change the GFAP subtype of the cerebellum, indicating that exhaustion exercise can induce reactive damage to astrocytes (De Souza et al., 2020). In addition, exercise inhibits microglia activation by regulating the toll-like receptor (TLRs) signaling pathway, downregulating the secretion of inflammatory factors such as TNF-α, C3, and IL-1β by pro-inflammatory microglia induced pro-inflammatory astrocytes, and alleviates neuroinflammation and cascade immune responses in animal brains (Liddelow et al., 2017; Ma et al., 2013). Other studies have also confirmed that low-intensity rotational exercise intervention can significantly reduce the expression level of C3 protein, a marker of pro-inflammatory astrocytes, in the hippocampus of mice (Nakanishi et al., 2021). A 6-month running intervention downregulated the levels of pro-inflammatory type astrocyte markers (Serping1, Srgn) in the hippocampus of AD model mice, while increasing the levels of anti-inflammatory type astrocyte markers (S100A10) (Belaya et al., 2020). These changes are accompanied by a decrease in inflammation levels within the AD brain and improvement in cognitive impairment. Continuous aerobic exercise for 8 weeks (12 m/min, 60 min/time, 5 times/week) inhibited the NF-κB signaling pathway in non-alcoholic fatty liver disease in mice and downregulated the expression levels of the liver inflammatory factors TNF-α and IL-6 (Jiang et al., 2024). Comassi et al. (2018) found that the expression of NLRP3, caspase-1, and IL-1β mRNA in the serum of middle-aged individuals was significantly downregulated after acute exercise. Wang et al. (2019) found that 4 weeks of aerobic exercise can inhibit the acetylation of forkhead box transcription factor O1 (FOXO1) and promote the phosphorylation of FOXO1 in the brain tissue of diabetes rats to inhibit the activity of FOXO1, thereby inhibiting the acetylation of NF-κB, reducing its transcriptional activity, and down regulating the protein expression of TNF-α, NLRP3, and IL-1β, thereby inhibiting the activation of NLRP3 inflammasome. In addition, 12 weeks of aerobic exercise upregulated the expression of irisin in the hippocampus of mice with post-stroke depression and inhibited the production and accumulation of NF-κ B. The protein expression of NLRP3, caspase-1, and IL-1β mRNA is significantly reduced, thereby inhibiting the inflammatory response (Tang et al., 2022).

At present, there are few reports on the regulation of astrocyte mediated neuroinflammation by exercise, most of which are limited to animal behavioral analysis and detection of targeted protein components. Further research is needed to determine the most suitable exercise methods or combination exercise regimens. Using *in vivo* and *ex vivo* multi-channel techniques for electrical signal analysis, we aimed to verify that motor regulation of astrocyte mediated neuroinflammation is a promising therapeutic target for the prevention and treatment of Parkinson’s disease.

## Exercise and PD prevention and treatment

Exercise, as a form of physical activity, can improve muscle coordination and exercise activity in patients with PD (Llamas-Velasco et al., 2021). Clinical studies and epidemiological pathology have shown that physical therapies such as aerobic, balance, and physical and mental exercise are both feasible and effective in PD rehabilitation, significantly alleviating behavioral disorders such as gait disorders, postural instability, and bradykinesia in PD patients, and improving their quality of life (Deuel and Seeberger, 2020). In addition, exercise enhances brain network function of PD patients and reduces inflammatory energy metabolism.

### Aerobic exercise and PD exercise prevention and treatment

Aerobic exercise is considered an important adjunctive therapy to pharmacotherapy in the clinical management of Parkinson’s disease, showing immediate benefits in improving motor dysfunction and learning ability in patients with PD, and a 6-month brisk walking and balance program can alleviate motor symptoms, promote functional and gait performance, walking ability, and dynamic balance in patients with mild to moderate PD (Mak and Wong-Yu, 2021). Table tennis exercise programs have potential safety and efficacy in improving activities of daily living (ADL) and motor symptoms in PD patients (Inoue et al., 2020). In addition, a 6-week Nordic walking training program can improve functional performance, gait quality, and quality of life of PD patients, and demonstrate the same effects as standard rehabilitation (Szefler-Derela et al., 2020). They showed that PD patients showed significant improvement in their training actions and daily activity performance after training (Bek et al., 2021) (as shown in Table 2).

**TABLE 2:** Aerobic exercise and PD exercise prevention and treatment.

Authors	Sample size	Age	Experimental group	Control group	Main result
Mak and Wong-Yu (2021)	BW (33) CON (31)	≥ 30	Fast walking group (fast walking and balance): (150 min/week, 2-3 times/week, RPE score: 11∼15, 40∼60% Hrmax, gradually transition from 60 to 150 min/week within 6 weeks)	Control group receives upper body training and does self-practice 2-3 times a week.	Compared to control group, BW had a larger and significant decline in Movement Disorder Society—Unified Parkinson’s Disease Rating Scale (MDS-UPDRS) exercise scores, and showed significant improvements over baseline in timed wake up time (TUG), fast walking speed (FGS), 6-min walking distance (MWD), and Mini-BEST Evaluation System test (Mini-BEST) scores.
Inoue et al. (2020)	PD patients (male 7, female 2)	71.8 ± 7.2	Table tennis: 40∼60% HRmax RPE (75 min, 1 time/week, RPE score: 11∼15, 75 min can be divided into morning and afternoon; Warm up and tidy up before and after exercise)	Before the study, eight patients received oral medication and one was treated with DBS without oral medication, so the patients performed physical activity and had no additional physical activity for 6 months.	MDS-UPDRS Part II (motor experience of daily living) and Part III (motor examination) improved at 3 and 6 months, while the MDS-UPDRS Part I total score (non-motor experience of daily living) and IV (motor complications) were unchanged. Montreal Cognitive Assessment (MOCA), Frontal Assessment Battery (FAB), Self-Rating Depression Scale (SDS) and Self-Rating Anxiety Scale (SAS) remain unchanged. A Bonferroni type multiple comparative analysis of MDS-UPDRS Part II showed improvements in the molecular counts of speech (baseline to 3 months) and getting up, riding, or sitting in a deep chair (baseline to 6 months), while there were no significant differences between MDS-UPDRS Part I and Part IV.
Szefler-Derela et al. (2020) ^]^	NW (20) SR (20)	50 75, mean age 60	NW (performed in an outdoor park): (12 rehabilitation sessions, 90 min/time, once/2 weeks, for 6 weeks). The warm-up portion(5-10 min) and specialized NW training (60 min), relaxation and stretching(5-10 min) were designed to improve walking intensity and walking distance in PD patients.	The SR rehabilitation facility takes place indoors, and classes last 45 min and include tailored, standard, generic exercises designed to improve fine motor and gross motor skills (stretching, high-amplitude movement), as well as exercises such as muscle strength, flexibility, and balance.	After 6 weeks of training, the median UPDRS (including bradykinesia, muscle rigidity, and balance scores), Dynamic Gait Index (DGI), TUG, and Parkinson’s Disease Questionnaire-39 (PDQ-39) of NW and SR improved significantly. The reduction in dysfunction measured by UPDRS Part III scores was significantly greater in NW than in SR. Gait quality and balance control were improved by DGI scores compared with MDC scores. The reduction in gait disorders measured by DGI scores in NW was greater than in SR, and the risk of falls decreased in both groups. TUG test results improved slightly in both groups, and functional improvements in both rehabilitation groups were associated with significant changes in the PDQ-39 measured quality of life score.
Bek et al. (2021)	experiment: (AO + MI) (6) Control (4)	47 73	150 min/time, 5 movements (three individual movements and 2 core movements for 6 weeks), can also be Specified according to personal preference (such as 25 min/time, 1 time/day, lasting 6 days or 25 min/time, 2 times/day, lasting 3 days). Initial testing: Each patient selected three “personal” actions that promised improvement (such as QQ, writing, and opening and closing food containers), in addition to engaging in regular daily tasks (handling silver coins and sorting train tickets).	The control group received regular treatment and a weekly telephone interview to keep in touch. Performance in individual training and untraining as well as core actions was also assessed, with participants viewing videos from a third- Person perspective while engaging in kinesthetic images and then performing actions (3/actions, 24 experiments).	DextQ-24 was used before and after the intervention to assess dexterity in daily tasks, PDQ-39 was used to assess quality of life, kinaesthetic and visual imagery Questionnaire, KVIQ asks participants to perform physical acts and then imagine performing simple movements involving different body parts (upper limbs, lower limbs, trunk, shoulders, and head). Visual and kinesthetic subscales are used to assess the vividness of the image and the intensity of the sensation, respectively. After the intervention of PD patients, PDQ-39 and KVIQ showed no significant improvement, self-reported flexibility and simplicity, and selective response time were improved, and the completion time of individual trained and untrained movements was shortened in the intervention group, and all types of difficulty grades were reduced, while the control group showed no improvement in completion time or difficulty grades.

### Physical and mental exercise and PD prevention and treatment

Physical and mental exercise is the traditional form of exercise in China. Different physical and mental exercise programs improve PD in terms of strength, flexibility, and range of motion, and reduce motor delay and muscle stiffness in PD patients. The results showed that somatosensory play combined with Wuqinxi training could improve balance, frozen gait, cognitive function, anxiety and depression in PD patients with dyskinesia, thereby improving their quality of life (Shen et al., 2021). Long term Tai Chi training can improve motor function, especially gait and balance, in PD patients. Enhancing brain network function, reducing inflammation, improving amino acid, energy, and neurotransmitter metabolism, as well as reducing vulnerability to dopaminergic degeneration, may be the mechanisms underlying the effectiveness of Tai Chi training (Li et al., 2022). Yoga is superior to proprioceptive training in improving balance and proprioceptive sensitivity in PD patients (Cherup et al., 2021). Study confirmed, continuous clinical Pilates exercise for 8 weeks has a positive impact on balance, functional activity, lower limb strength, and fall risk in PD patients (Çoban et al., 2021). Dance training has some motor and non-motor benefits for PD patients, manifested in significant effects on their balance, gait, executive function, and positive quality of life (Duarte et al., 2023; as shown in Table 3).

**TABLE 3:** Physical and mental exercise and PD exercise prevention and treatment.

Authors	Sample size	Age	Experiment group	Control group	Main resolute
Shen et al. (2021)	Experiment group (50) control group (50)	≥ 18	Experimental group: 1∼2 weeks of motion- sensing games combined with Wuqin play training (20∼30 min/time, 3 times/week) 3-8 weeks combined with traditional Chinese medicine Five poultry play training (40 min/time, 3 times/week), in the order of tiger, deer, bear, ape, crane training, each play is divided into two movements, a total of 10 types. Divided into potential breath, tiger play: tiger lift, tiger flutter; Deer play: deer arriving, deer running; Bear play: Bear luck, bear shake; Ape play: ape raising, ape picking; Crane play: crane stretching, crane flying; Close the potential, wire turn to the yuan.	The control group received conventional training methods. Exercise appropriately according to the living habits of patients, and give routine health education such as diet, fall prevention, posture balance, daily life and emotional regulation.	After 8 weeks of intervention, the UPDRS-III score of control group was lower than before intervention; There was no significant difference between Berg Balance Scale (BBS) score and Freezing of Gait Questionnaire (FOGQ) score before and after intervention. The UPDRS-III and FOGQ scores of observation group were lower than before intervention and lower than control group, and the BBS scores were higher than before intervention and higher than control group. HAMA, HAMD and PDQ-39 scores in the observation group were lower than before intervention and control group, and MoCA scores were higher than before intervention and control group. The Hamilton Anxiety Scale (HAMA), Hamilton Depression Scale (HAMD) scores of the control group were lower than those before intervention, but PDQ-39 scores and MoCA scores had no significant differences compared with those before intervention.
Li et al. (2022)	Tai Chi group (32) fast walking group (31) Inactive group (32)	50 80	Tai Chi group: (60 min/time, 2 times/week, 12 months) Fast walking group: (60 min/time, twice/week, 12 months, 50∼60% HR _max_)	Inactive group: No intervention was given	PD patients showed significant improvement in Berg Balance Scale (BBS) scores, UPDRS, Time Up and Go test (TUG) and 3D gait analysis assessment. Compared with control group and fast walking group, Taijiquan group significantly improved the step width of PD patients at 6 and 12 months.
Cherup et al. (2021)	YoMed (21) PRO (12)	70.6 ± 9.9	YoMed:(45 min/time, twice/week, 12 weeks), the patient sat with the eyes in a meditation position closed, including simple sitting, mountain standing, downward dog, warrior I, warrior II, wind tree pose, cobra pose, skylark pose, ostrich pose, triangle pose and mountain sitting posture 17 practice positions.	PRO:(45 min/time, 2 times/week, 12 weeks), walking on uneven surfaces and balancing on specific equipment, including a semicircle balance ball and an aerobic ball.	The YoMed program produced greater improvements on the Tinetti Balance Assessment (TIN) and JK, and there was also a trend improvement in TUG scores. The dynamic balance objective test (DMA) score decreased significantly, while the total residence TIME (TIME) on the dynamic balance test platform increased significantly. In addition, YoMed intervention had large in-group effect sizes for FES, BESS, TIN, DMA, TIME, TUG, and jkflex. The in-group effect size of the PRO program was limited only to DMA and TIME, UPDRS improvements.
Duarte et al. (2023)	Dance movement group (13)	65.9 ± 6.5	Dance exercise group (50 min/time, 2 times/week, for 6 months) Dance styles include: Tango, ballroom dance, city dance, Samba, ballet, modern dance and local dance. Corresponds to the neuropsychological functions that are affected by PD: cognitive, sensory, motor, emotional, and social aspects.	No control group	The Performance Oriented Mobility Assessment (POMA) tested for significant improvements in movement (balance and gait) and the Frontal Assessment Battery (Frontal Assessment Battery). FAB) showed significant improvement in test executive function, Montgomery-Asberg Depression Rating Scale (Montgomery-Asberg Depression Rating Scale, The MADRS test assessed a significant reduction in depressive symptoms and a significant increase in PDQ-39 quality of life after the intervention. MDS-UPDRS TOTAL) severity decreased significantly.

### Balance training and PD exercise prevention and treatment

Balance training relies on interacting several physiological systems (musculoskeletal, neuromuscular, cognitive, and sensory systems) with environmental factors and task execution. Different balance training methods can improve or maintain the balance and gait performance of elderly people with Parkinson’s disease. Sixteen week high-intensity tandem cycling intervention can improve the motor function and biochemical and functional neuroimaging variables of PD patients (Segura et al., 2020). In addition, it has also been demonstrated, anti-gravity treadmills showed improvements in gait freezing questionnaire (FOG) and activity ability in PD patients (Baizabal-Carvallo et al., 2020). Boxing training programs can evaluate the therapeutic effects of interventions on PD patients, particularly in terms of disease progression outcomes (Blacker et al., 2024). Ten weeks of table tennis exercise had the potential to improve balance control, mental health, and self-physical activity levels in PD patients (Olsson et al., 2020; as shown in Table 4).

**TABLE 4:** Balance training and PD exercise prevention and treatment.

Authors	Sample size	Age	Experiment group	Control group	Main result
Segura et al. (2020)	Intervention group (3 males and 4 females) control group (4 males and 2 females)	≤ 65	Intervention group: tandem bicycle: adjustment stage: 30∼40 min, the first 4 weeks (1 time/week), the last 4 weeks (2 times/week), warm-up for 5 min (30∼40 rpm for low resistance treadling), then 20-50% of HR (40∼60 rpm for 60 min), and finally relax for 5 min (30∼40 rpm for 60 min). High-intensity tandem riding: 16% of HR (≥ 80 rpm) (30∼60 min/time, 3 times/week, 16 consecutive weeks), warm-up for 10 min (30∼40 rpm for low resistance treadle), then ride at 80% of maximum heart rate for 20 min, relax for 5 min (30∼40 rpm). Do 10 min stretching exercises at the end of each session.	Adhere to conventional medication treatment	After 16 weeks, it was found that compared with the control group, the Unified Parkinson’s Disease Rating Scale (UPDRS), anthropometry, VO2 maximum value, PD biomarkers, and functional magnetic resonance imaging significantly increased. However, Weight and BMI, there was no difference in waist circumference between the groups.
Baizabal-Carvallo et al. (2020)	19 PD patients (10 males, 9 females)	72.68 ± 10.12	Instruct the patient to take the anti-PD medication at least 1-2 before each training session so that it is performed while the medication is “on.” Therapeutic anti-gravity treadmill training with M320 anti-gravity treadmill (60 min/time, 2 times/week, 4 consecutive weeks)	The control group was not given any intervention	The TUG test evaluates the improvement of mobility, dynamic balance, and fall risk in PD patients, while the Clinical Global Impression Scale (CGIS) assesses the severity of gait disorders at baseline and the improvement after the training course.
Blacker et al. (2024)	10 PD patients (6 males and 4 females)	average age 40	Boxing training: (60 min/time, 3 times/week for 15 consecutive weeks), each workout includes a warm-up, multiple rounds of non-contact boxing using training equipment and active rest. Boxer Development: Focus on training techniques Boxer Aerobics: Increased intensity, including high-intensity interval training Boxer Brain: Dual task training focused on cognitive challenges Training Module: Boxing Development (focused on training techniques) Boxer aerobics: Increased intensity (including high-intensity interval training); Boxer Brain Training: (focuses on cognitively challenging dual task training).	The control group was not given any intervention	Among the 10 PD subjects, 9 showed improvement in UPDRS motor scores, and training module 3 increased PD patients’ cognitive ability, reduced fatigue, and improved sleep quality. Clinical outcomes: Safety (adverse events), training intensity (using heart rate and perceived fatigue testing), tolerance (pain, fatigue, and sleep scores), as well as the Unified Parkinson’s Rating Scale (UPDRS-III), Perceived Fatigue Rate (RPE), Heart Rate (HR), and Perceived Mental Fatigue Monitor (RPME) before and after the project
Olsson et al. (2020)	PD patients (5 males, 4 females)	average age 66.9	Table tennis (120 min/time, 2 times/week (Friday and Sunday 16:00–18:00) for 10 consecutive weeks), including warm-up, instruction and a 10 min break. Training requirements require individual adjustments, including posture and footwork exercises (i.e., “left and right” and “in and out”), basic ping-pong stroke skills and ball control exercises, such as controlling the direction and force of the shot or playing with the non-dominant hand. Basic table tennis skills from beginners to leisure time playing experience etc.	No control group	After table tennis training, calculate the increase in gait speed through a 10 meter walking test; The score increases or remains unchanged in the Mini Best evaluation system test; Except for one subject, the Montgomery Osberg Depression Rating Scale (MADRS) scores were all low; The Frandin Cremby Activity Scale showed an increase in physical activity among all participants except for one. There was no significant change in other evaluation indicators, but the quality of life questionnaires (PDQ-8 and EQ-5D-3L) showed a trend in volume.

In summary, epidemiological data suggests that exercise can reduce the risk of developing PD and slow down its progression. Clinical evidence suggests that exercise is one of the most widely used non-pharmacological complementary therapies for PD, which is beneficial for the motor and cognitive function of PD patients. Therefore, detailed exercise prescriptions can be developed based on the specific situation of PD patients.

## The role of exercise regulated pro-inflammatory astrocytes in the prevention and treatment of Parkinson’s disease

Under PD pathological conditions, astrocytes can transform into pro-inflammatory and neurotoxic reactive astrocytes through morphological, molecular, and functional changes. Exercise can transform pro-inflammatory and neurotoxic reactive astrocytes into anti-inflammatory and neuroprotective reactive astrocytes, thereby reducing neuroinflammation and promoting neuronal survival in PD. Autopsy findings in PD patients show that physical activity can downregulate the expression of GFAP and MAP2 in the brain of PD patients, upregulate the expression level of TH protein, inhibit astrocyte proliferation and α-syn aggregation, which may be one of the mechanisms by which physical activity improves the expression and behavioral performance of substantia nigra TH in PD patients (Real et al., 2021). Research has found that 10 weeks of treadmill training can effectively inhibit the pro-inflammatory response of astrocytes in the substantia nigra and striatum of MPTP-induced PD model mice, upregulate the protein expression of BDNF, and inhibit the inflammatory response in astrocytes in the substantia nigra and striatum of PD model mice (Real et al., 2021). Four week moderate-intensity treadmill training can significantly downregulate the expression level of glial fibrillary acidic protein in the striatum of 6-Hydroxydopamine (6-OHDA) unilaterally damaged PD model rats, reduce the number of rotations induced by APO, increase the number of left forelimb uses in cylinder tests, significantly increase the total number of steps in grid experiments, and enhance autonomous activity (Shi et al., 2017). A study showed that 4-week autonomous running wheel exercise can significantly reduce the number of glial fibrillary acidic protein positive cells (GFAP^+^) in the substantia nigra striatum of MPTP model mice, downregulate the co-expression level of S100 β/GFAP+, significantly reduce the number of Iba-1 + cells, and significantly increase the number of substantia nigra TH positive cells and striatal TH positive fiber terminal content (Gil-Martínez et al., 2018). In addition, investigated the effects of treadmill training on motor deficits and glial fibrillary acidic protein (GFAP) expression in PD model rats. The results showed that 4-week treadmill training significantly reduced the number of rotations in PD model rats, shortened the latency period of crossing the balance beam, and significantly downregulated the expression level of glial fibrillary acidic protein in the striatum (Dutra et al., 2012). 4-week incremental intensity treadmill training can inhibit the activation of reactive astrocytes in the cerebellum of rotenone induced PD model rats, significantly reduce the optical density of glial fibrillary acidic protein (GFAP) immunopositive cells, significantly reduce the number of apoptotic Purkinje cells in the cerebellum, and significantly improve motor coordination and balance (Lee et al., 2018). Furthermore,4-week high-intensity (18 m/min, 40 min/day, 5-day/week) endurance training significantly downregulated the expression levels of glial fibrillary acidic protein (GFAP), calcium binding protein (S100B), and neuron specific enolase (NSE) in the striatum of MPTP model mice (Al-Jarrah and Jamous, 2011). Other studies have also shown that 4-week treadmill training can significantly reduce the optical density of CD-11c/b and GFAP in the striatum of 6-OHDA damaged PD model mice, significantly decrease the number of inducible nitric oxide synthase positive cells, significantly downregulate the protein expression levels of GFAP and CD-11c/b, and significantly increase the content of TH immune positive fiber terminals in the striatum (Real et al., 2017). Some scholars have studied further the effects of different exercise modes and intensities on the physiological changes of striatal and ventral midbrain glial cells in PD model rats. The results showed that high-intensity interval exercise (HIE) could increase the activity of anti-inflammatory reactive astrocytes in the striatum directly involved in exercise control, thereby producing neuroprotective effects. And it was found that both HIE and combined exercise (CE) increased the expression of dopamine transporters (DAT) in the striatum, significantly reducing the loss of dopamine neurons in the ventral midbrain (VM) (Jo et al., 2025). Although the above studies only explored the effects of exercise on central reactive glial cells in PD model animals, they did not simultaneously detect the effects of exercise on neuroinflammatory factors. However, research has confirmed that astrocytes are key glial cells involved in the regulation of neuroinflammation. When faced with pathological stimuli such as inflammation, trauma, and neurodegeneration, they reshape their morphology, genome, metabolism, and functional characteristics to form reactive astrocytes, manifested as hypertrophy, release of inflammatory mediators and nutritional factors, increased expression of glial fibrillary acidic protein and vimentin, which are common in neurodegenerative diseases such as PD.

In summary, on the one hand, exercise can prevent and treat PD by inhibiting the pro-inflammatory activation mediated by astrocytes. On the other hand, exercise can alleviate neuroinflammation, improve astrocyte immune function, and reduce self-cytotoxicity by inhibiting astrocyte subtypes, which is beneficial for PD prevention and control. Currently, most literature reports on the prevention and control of neuroinflammation mediated by exercise regulated microglia and neuroinflammation mediated by exercise regulated astrocytes to prevent and treat other neurodegenerative diseases such as AD, there is still limited literature on the prevention and treatment of Parkinson’s disease through exercise regulation of astrocytes mediated neuropathy. However, astrocytes are the most widely distributed in the mammalian brain, capable of responding to inflammatory signals and regulating inflammation, participating in the regulation of multiple life processes of the nervous system in physiological and pathological states. Combined with exercise and astrocyte mediated neuroinflammation, as well as exercise and PD, it can be inferred that exercise regulation of astrocyte mediated neuroinflammation may be a potential target for PD treatment.

## Summary and outlook

### Summary

Neurotoxic reactive astrocytes are closely related to PD pathophysiology as mediated by neuroinflammtion. Directly preventing the transformation of astrocytes into neurotoxic phenotypes has neuroprotective effects and can prevent the loss of DA neurons and behavioral defects in PD model mice. Exercise can reduce the number of central pro-inflammatory and neurotoxic reactive astrocytes, and increase the number of anti-inflammatory and neuroprotective reactive astrocytes. Various types of exercise therapy are widely used as adjunctive therapy for drug therapy in PD patients, and their potential mechanisms are closely related to the conversion of exercise-induced pro-inflammatory and neurotoxic reactive astrocytes to anti-inflammatory and neuroprotective astrocytes. Blocking the function of reactive astrocytes and increasing their beneficial effects is a potential strategy for preventing and treating the pathological progression of PD and related behavioral disorders.

### Outlook

This article mainly focuses on the perspective of neuroinflammation mediated by neurotoxic reactive astrocytes, explores the potential role of exercise in the prevention and treatment of PD, and summarizes the current research progress. However, there are still many evidence gaps that need further clarification: (1) Based on the wide sources of pro-inflammatory cytokines (such as astrocytes, microglia, and damaged neurons), the mechanism of changes in pro-inflammatory cytokines in exercise intervention still needs further confirmation; (2) The mechanism by which exercise regulates the anti-inflammatory effect on astrocytes still needs to be elucidated; (3) At present, most of the research on exercise intervention for PD patients is literature on improving motor dysfunction, and most of the related mechanism research comes from animal studies, which still needs to be devoted to human research; (4) In the prevention and control of PD mediated by exercise regulation of astrocytes, it is necessary to explore more possible mechanisms as potential targets for the treatment of PD, which is also one of the urgent problems to be solved in basic and applied research. Therefore, in the prevention and treatment of PD, future efforts should be focused on pathological detection of brain tissue in PD patients, and precise and effective exercise programs, combined exercise interventions (such as aerobic exercise + resistance exercise), and combined intervention methods (such as nutritional supplements + exercise supplements) may be needed to better regulate astrocyte mediated neuroinflammation in PD patients and improve motor dysfunction. The solution to the above problems will provide reference for finding effective measures to prevent and control PD.
